# Provision of information by midwives for pregnant women in England on guidance on foods/drinks to avoid or limit

**DOI:** 10.1186/s12884-023-05441-8

**Published:** 2023-03-08

**Authors:** Lucy Beasant, Jenny Ingram, Rachel Tonks, Caroline M. Taylor

**Affiliations:** 1 Centre for Academic Child Health, Bristol Medical School, University of Bristol, Canynge Hall, 39 Whatley Road, Bristol, BS8 2PS UK; 2 School of Health and Social Wellbeing, University of the West of England, Bristol, UK

**Keywords:** Pregnancy, Diet, Nutrition guidance, Public health nutrition, Midwife, PEAR Study

## Abstract

**Background:**

The National Health Service (NHS) website gives guidance for pregnant women in England on foods/drinks to avoid or limit because of microbiological, toxicological or teratogenic hazards. These include, for example, some types of soft cheeses, fish/seafood and meat products. This website and midwives are trusted sources of information for pregnant women, but the ways in which midwives can be supported to provide clear and accurate information are unknown.

**Aims:**

The aims were to: (1) determine midwives’ accuracy of recall of information and confidence in delivering the guidance to women; (2) identify barriers to provision; (3) identify the ways in which midwives provide this information to women.

**Methods:**

Registered Midwives practicing in England completed an online questionnaire. Questions included those on what information they provided and their confidence in delivering it, the ways they provided information on foods to avoid/limit, their recall of some of the guidance, and what resources they used. Ethics approval was given by the University of Bristol.

**Results:**

More than 10% of midwives (*n* = 122) were ‘Not at all confident/Don't know’ in providing advice about ten items, including game meat/gamebirds (42% and 43%, respectively), herbal teas (14%) and cured meats (12%). Only 32% correctly recalled overall advice on eating fish, and only 38% the advice on tinned tuna. The main barriers to provision were lack of time in appointments and lack of training. The most usual methods of disseminating information were verbal (79%) and signposting to websites (55%).

**Conclusion:**

Midwives were often unconfident about their ability to provide accurate guidance, and recall on items tested was frequently mistaken. Delivery of guidance by midwives on foods to avoid or limit needs to be supported by appropriate training and access to resources, and sufficient time in appointments. Further research on barriers to the delivery and implementation of the NHS guidance is needed.

## Introduction

The guidance given to pregnant women in England is to follow a healthy diet broadly similar to that advised for the general population [1]. There is additional guidance on several items of foods and drinks that pregnant women are advised to either limit or avoid consumption altogether [2–9]. This guidance is based on several factors as follows. (1) Exposure to toxic metals and pollutants such as mercury, lead, dioxins and polychlorinated biphenyls (e.g. from some types of fish, game meat/gamebirds) is associated with a risk of adverse developmental effects including neurodevelopmental disorders [10–13]. (2) Microbiological hazards such as listeria, toxoplasmosis and salmonella (e.g. from unpasteurised milk, some types of soft cheese, uncooked cured meats) can lead to miscarriage, premature birth and stillbirth [14–16]. (3) Excess provision of vitamin A (e.g. in liver and liver products) can cause teratogenesis [17]. (4) Pharmacological actions or interactions with drugs can be caused by some types of herbal teas, including fennel, ginger, chamomile and peppermint [18]. For example, ginger and chamomile may enhance the effect of central nervous system depressants such as clonazepam, and the tannins in some herbs can interfere with the absorption of iron with a potential impact on the prevalence and severity of anaemia. Non-compliance with guidance on foods and drinks to avoid or limit in pregnancy can have serious consequences: in 2019, for example, pregnancy-associated cases of listeria accounted for 18% of all cases and one-third of these cases resulted in stillbirth or miscarriage in England and Wales [19].

Little is known about the midwife’s role in providing nutrition education as distinct from lifestyle promotion. Studies on nutrition guidance in pregnancy have generally focused on healthy eating guidance, diet quality and weight management [20–22], or on a particular age group [23] or food item (e.g. fish [24]), with the few studies on specific foods to avoid or limit mainly focussed only on listeria [25, 26]. Both women’s knowledge of guidance and adherence appear to be limited: a study of pregnant and recently pregnant women in Australia found that women’s knowledge of foods to avoid was poor (93% incorrectly identified at least one unsafe food as safe to eat) [27] and in Canada only 53% of a group of pregnant women made dietary changes to follow food safety recommendations [28]. We have previously reported that adherence to the guidance by women resident in England during pregnancy is generally good, but there are some items for which it is not: herbal tea, gamebirds and game meat, cured meats, soft cheese and standard multivitamins [29]. However, there are no previous reports to our knowledge on the role of midwives or other healthcare professionals in advising women and supporting their choices on foods/drinks to avoid or limit in pregnancy in England.

Guidance on foods and drinks to avoid or limit in pregnancy is embedded in the National Institute for Health and Care Excellence (NICE) Quality Standards [30] and is derived from a number of expert reports (e.g. Scientific Advisory Committee on Nutrition (SACN) *Advice on fish consumption: benefits and risks* [31]). The guidance is promoted on the National Health Service (NHS) website page for England [3], and information is also available through a range of leaflets and apps, and on other websites hosted by charities and commercial organisations (for example [32]). Provision of this information in a way that is readily accessible and easily understood by women is essential in order to minimise or eliminate exposures to toxic metals and pollutants, microbiological hazards, teratological substances, and pharmacological interactions or actions. Midwives are highly trusted sources of information for pregnant women in England [33] and it is crucial that they are enabled to provide information on the guidance confidently, effectively and accurately, whether this is directly or by signposting to other sources of information. The aims of this study were to determine: (1) how confident midwives are in their ability to deliver the guidance to women and the accuracy of midwives’ recall of information; (2) the barriers to optimal provision of information; (3) the ways in which midwives provide information on the guidance on foods and drinks to avoid or limit to women and what resources they use. This was intended to inform the possible need for additional training and resources on the guidance.

## Methods

The study is part of a larger mixed methods study on dietary exposure to toxic metals in pregnancy (Pregnancy, Environment And nutRition (PEAR) Study) [34]. Registered Midwives (RM) practising in England and delivering antenatal care in the previous 2 years were invited to complete a custom-designed online questionnaire hosted on Jisc Online Surveys (version available in 2021) [35]. Ethics approval was given by the University of Bristol Health Sciences Research Ethics Committee (reference 106,742, 21 April 2021). The primary purpose of the PEAR Study is to investigate guidance on foods that contribute to exposure to toxic metals (mercury and lead) in pregnancy within the context of foods and drinks to avoid or limit more generally.

The initial version of the questionnaire was tested with registered midwives who practiced in England and had delivered antenatal care in the previous 2 years in an adapted ‘Think Aloud’ exercise, and was modified according to their feedback [36]. Participants (*n* = 7) were emailed a link to access the electronic questionnaire and answered each question in the presence of a researcher (LB). ‘Think Aloud’ discussions were conducted remotely via video or telephone call and were recorded using an encrypted digital audio-recorder. Participants were asked to ‘Think Aloud’ as they accessed and filled in the questionnaire, vocalising their thoughts about the questions, covering, for example, any comprehension issues, the acceptability of available answers and technical problems including skip rules and the order of questions. Three ‘practice questions’ were provided at the beginning of the questionnaire to ensure the participant understood what the exercise involved. Questions and queries from the participant were addressed by the researcher, who made brief field notes during the exercise and remained silent other than a polite reminder to the participant to ‘keep thinking aloud’ if they fell silent. When the participant had completed the questionnaire, the researcher used notes made during the exercise to probe any area where the participant seemed uncertain. Problems identified were categorised into: (1) Comprehension (e.g. any misunderstanding of a word, phrase or response option; (2) Retrieval (e.g. a recall problem); (3) Judgement (e.g. recalled experiences are irrelevant or inadequate); (4) Response (e.g. participant’s response is inconsistent with the personal experience expressed or the desired response is missing from the response choices. Changes made included: adding additional response options to questions (e.g.*, Question 15 ‘What are the main ways that you provide women with information on healthy eating during pregnancy?’* *and Question 16* ‘*What are the main ways that you provide* *information on specific foods to limit or avoid during pregnancy?’ the response option ‘Maternity handheld records’ was added*), correcting technical problems with accessing sub-questions (e.g.*, Question 26 ‘Are you able to provide the service that you would ideally like to in giving information on diet to pregnant women?’ incorrectly allowed participants to select more than one response, this was converted to a single response answer*), and changes to the wording of a small number of questions and response options (e.g.*, Question 18.13 originally stated ‘Fresh tuna’ and was changed to ‘Fresh tuna steak’)*. Development of the questionnaire was iterative, with alterations being made in response to the comments of up to five participants at a time, until data saturation was reached and no new issues were reported.

The finalised questionnaire was open from April 2021 to December 2021. Eligible participants were RM practising in England who had regularly provided antenatal care within the last 2 years. The questionnaire comprised 24 main questions, taking about 10–15 minutes to complete: 10 of these questions included clusters of sub-questions (e.g. the question ‘How confident would you be in providing advice to a pregnant women about each of the following drinks if asked during a routine appointment?’ included a list of five drinks or types of drinks and five possible responses for each in a grid pattern). Others included sub-questions that opened if the participant selected specific responses (e.g. for the response to ‘Nowadays, do you follow a particular diet?’, if the participant answered ‘Yes’ then a sub-question opened to ask about which particular diet they followed).

Participants were recruited primarily through publicity on the Royal College of Midwives website, professional networks, snowballing, Twitter adverts, and with paid advertising boosts on a study Facebook page linked to the study website (with direct access to the questionnaire from the study website [34]). Informed consent to participate was assured by completion of the questionnaire. With the exception of the screening questions to determine eligibility, no questions were compulsory to maximise the completion rate. Participants were able to re-access their partially completed questionnaire so that they did not have to complete it in one session. Questions included those in the following categories.

1. Screening questions (Confirmation of being an RM practising in England; Confirmation of regularly providing antenatal care within the last 2 years). Potential participants who did not pass the screening questions were not able to access the questionnaire.
2. Demographics (e.g. how many years worked as a RM, NHS Agenda for Change (AfC) band (rate of pay [37]), number of hours worked per week, qualifications, setting that they worked in, whether they had run antenatal classes in the last year, additional training in nutrition/diet, age, geographical location in England, ethnicity, whether they followed a particular diet).
3. Confidence in ability to provide information about the guidance to women (pate (meat and vegetarian), liver/liver products, uncooked cured meats, game meat and gamebirds, soft cheeses, unpasteurised milk, white fish, shark/marlin swordfish, oily fish, tinned and fresh tuna, sushi, shellfish, eggs, peanuts, standard multivitamins, omega-3 supplements, alcohol, caffeinated drinks and herbal teas; guidance on food to avoid involving cooking methods were not included: raw or partly cooked meat, washing or peeling of fruit and vegetables)
4. Recall of guidance on specific foods (those related to exposures to toxic metals: total fish, shark/marlin/swordfish, oily fish, tinned and fresh tuna, gamebirds and game meat)
5. Barriers to provision of information on guidance to pregnant women
6. Methods of provision of information about the guidance to women (e.g. verbal, leaflet, signposting to NHS website, other websites or apps)

The data were analysed with IBM SPSS Statistics version 26 using summary and descriptive statistics including categorical percentages (proportions). Where participants were able to choose more than one response category (e.g. qualifications) proportions were calculated for each category independently so that percentages added up to more than 100%. For some analyses, response categories were combined (e.g. Confidence in providing advice on foods and drinks to avoid or limit: the five response categories were *Don’t know, Not at all confident, Fairly confident, Confident, Very confident* and these were merged into three categories of *Don’t know/Not at all confident, Fairly confident* and *Confident/Very confident* for ease of interpretation.

## Results

The questionnaire information page was accessed by 1421 respondents. Most (1219) did not progress beyond the information page. Eleven were screened out as ineligible. The survey was completed by 122 participants, giving a completion rate of 122/202 (60%) for those who progressed beyond the information page. The demographics of the participants are shown in Table 1.

**Table 1:** Demographics of midwives completing online questionnaire (*n* = 122)

Demographic	n (%)
Worked as an RM (years)	
0–10	64 (52%)
> 10–20	28 (23%)
> 20	30 (25%)
Current NHS AfC band^a^	
Band 5 or 6	96 (80%)
Band 7 or 8	24 (20%)
Hours of work	
Full time (37.5 hours/week)	57 (47%)
Part time (< 37.5 hours per week)	64 (53%)
Qualifications^b^	
Degree/MSc in Midwifery	103 (84%)
Diploma/Advance Diploma in Midwifery	15 (12%)
Certificate in Midwifery	12 (10%)
Other	6 (5%)
Main antenatal work setting	
Community setting	69 (57%)
Hospital	25 (21%)
Delivery suite	9 (7%)
Other	19 (16%)
Run antenatal classes in last year	
Yes	23 (19%)
No	98 (81%)
Additional training in nutrition/diet	
Yes	13 (11%)
No	108 (89%)
Age (years)	
> 50	30 (25%)
> 30–49	62 (51%)
< 30	30 (25%)
Geographical location of work in England	
North East/North West/Yorks and Humber	19 (16%)
East Midlands/West Midlands/East of England	5 (4%)
Central or Greater London/South East/South West	98 (80%)
Ethnicity (*n* = 119)	
White	113 (95%)
Other	6 (5%)
Special diet	
No	77 (63%)
Yes^b^	45 (37%)
Vegan/vegetarian/flexitarian	25 (56%)
Low carb/gluten or wheat free/Paleo or Atkins	9 (20%)
Other (dairy free, FODMAP, low calorie)	11 (24%)

Data included in ‘Other’ category due to participant number ≤ 5 for some categories

*RM* Registered Midwife

^a^AfC, Agenda for Change [payscales] 2022/3: Band 5/6, £27,055 to £40,588 per year; Band 7/8 £41,658 to £91,767 [37].

^b^Participants could choose more than one category

Most midwives were ‘Confident’ or ‘Very confident’ in their ability to deliver the correct advice on specific foods and drinks to avoid/limit during a routine appointment (> 90% ‘Confident/Very confident’ for eggs, liver/liver products, unpasteurised milk, alcohol) but there were some items for which this was not the case: for example > 10% were ‘Not at all confident/Don't know’ for gamebirds (43%), game meat (42%), omega-3 supplements (25%), herbal teas (14%), cured meats (12%), white fish (12%), peanuts (11%) and shellfish (12%) (Table 2).

**Table 2:** Midwives’ confidence in providing advice on foods and drinks to avoid or limit in a routine appointment and recall of advice on foods included for toxicological reasons (*n* = 122)

	NHS website guidance^a^		Examples included in questionnaire	Midwives’ confidence in providing advice on foods and drinks to avoid or limit in a routine appointment (***n*** = 122)			Recalled advice correctly^**d**^
	Guidance	Reason		Don’t know/Not at all confident	Fairly confident	Confident/ Very confident	
**Foods**							
Meat and meat products							
Pate (meat and vegetarian)	Avoid	Microbiological	–	4 (3%)	10 (8%)	108 (89%)	–
Liver/liver products	Avoid	Teratogenic	–	6 (5%)	5 (4%)	111 (91%)	–
Cured meats (uncooked)	Avoid	Microbiological	Salami, prosciutto, pepperoni, chorizo	14 (12%)	28 (23%)	80 (66%)	–
Game meat	Avoid	Toxicological	Venison/deer, hare, rabbit, wild boar	51 (42%)	29 (24%)	41 (34%)	31 (26%) (n = 121)
Gamebirds	Avoid	Toxicological	Grouse, goose, pheasant, partridge, pigeon	52 (43%)	29 (24%)	41 (34%)	38 (31%) (n = 121)
Raw or undercooked meat^b^	Avoid	Microbiological	–	–	–	–	–
Dairy products							
Soft cheese (uncooked mould-ripened soft cheese with a white coating, uncooked soft blue cheese, any soft cheese made with unpasteurised milk)	Avoid	Microbiological	Brie, Camembert, Danish blue, soft goat’s cheese	3 (3%)	9 (7%)	109 (90%)	–
Unpasteurised milk/cream	Avoid	Microbiological	–	5 (4%)	6 (5%)	111 (91%)	–
Fish							
White fish			Haddock, cod, pollock, tilapia, including breaded fish and fish fingers	14 (12%)	10 (8%)	98 (80%)	
Shark, marlin, swordfish	Avoid	Toxicological		11 (9%)	10 (8%)	101 (83%)	95 (79%) (*n* = 120)
Oily fish	Limit	Toxicological	Salmon, trout, mackerel, herring	9 (7%)	10 (8%)	103 (84%)	64 (53%) (n = 121)
Tuna (tinned)	Limit	Toxicological	–	6 (5%)	14 (12%)	102 (84%)	46 (38%) (n = 121)
Tuna (fresh)	Limit	Toxicological	–	13 (11%)	17 (14%)	92 (75%)	52 (43%) (n = 121)
Sushi made with raw fish	Avoid	Microbiological	–	14 (12%)	21 (17%)	86 (71%)	–
Smoked fish, uncooked^bc^	Avoid	Microbiological	–	–	–	–	–
Shellfish, uncooked	Avoid	Microbiological and toxicological	–	14 (12%)	19 (16%)	88 (72%)	–
Fruit, vegetables and salads							
Unwashed or unpeeled fruit, vegetables and salads^b^	Avoid	Microbiological Microbiological	–	–	–	–	–
Liquorice root^b^	Avoid		–	–	–	–	–
Eggs							
Raw or partly cooked hens’ eggs if not stamped with British Lion mark Raw or partly cooked goose, duck and quail eggs	Avoid	Microbiological	–	4 (3%)	7 (6%)	111 (91%)	–
Peanuts	Avoid only if nut allergy	Allergy		13 (11%)	19 (16%)	89 (73%)	–
**Supplements**							
Standard multivitamins containing vitamin A	Avoid	Teratogenic	–	10 (8%)	12 (10%)	100 (82%)	–
Omega-3 supplements derived from fish liver oil	Avoid	Teratogenic	–	31 (25%)	26 (21%)	64 (53%)	–
**Drinks**							
Alcohol	Avoid	Toxicological	Wine, beer, spirits, cider, alcopops, cocktails	2 (2%)	4 (3%)	116 (95%)	–
Caffeinated drinks	Limit	Toxicological					–
Tea/coffee			–	4 (3%)	14 (12%)	104 (85%)	
Soft drinks			–	6 (5%)	13 (11%)	103 (84%)	
Energy drinks			Red Bull, Monster	6 (5%)	13 (11%)	102 (84%)	
Herbal tea	Limit	Pharmacological	Ginger, raspberry, mint	17 (14%)	28 (23%)	77 (63%)	–

^a^NHS website: Foods to Avoid in Pregnancy [3]

^b^These items were not included in the questionnaire

^c^Added to the NHS website in summer 2022 after the study ran so not included in the study

^d^Included in questionnaire only for items related to exposures to toxic metals (mercury and lead)

With regard to recall of guidance on some specific items, however, 68% were not able to correctly recall the guidance for overall fish consumption, 47% for oily fish, 62% for tinned tuna (54% incorrectly thought that the advice was to eat up to two medium sized cans per week), 57% for fresh tuna, and 21% for shark/marlin/swordfish. Sixty-nine percent did not correctly recall the guidance on gamebirds and 74% on game meat.

Only 14 (12%) of the participants felt they could always deliver the service they would ‘ideally like in giving information on diet to pregnant women’ and 23 (19%) felt that they were never able to do this. Common reasons for prevention of delivery of an ideal service included lack of time in appointments (*n* = 81 (66%)), lack of training (*n* = 64 (58%)) and lack of suitable resources to be passed on (*n* = 51 (42%)).

The most prevalent method of midwives providing information on the guidance to women on foods and drinks to avoid or limit was verbal, followed by signposting to websites, primarily the NHS website, and to apps, primarily NHS Trust-specific apps (Table 3).

**Table 3:** Midwives’ methods of provision of information (*n* = maximum 122)

Methods	Information on general healthy eating	Information on foods/drinks to avoid or limit	Resources for information on foods/drinks to limit or avoid^**a**^	
**Verbal**	109 (89%)	96 (79%)		
**Information about websites**	66 (54%)	67 (55%)	NHS Choices Website	66 (99%)
			Start4Life	22 (33%)
			Online pregnancy book	17 (25%)
			Tommy’s	16 (23%)
			BBC Website/Mumsnet/Other	13 (19%)
**Maternity handheld records**^**b**^	59 (48%)	54 (44%)	–	–
**Information about apps**	40 (33%)	29 (24%)	Trust-specific app	23 (79%)
			Pregnancy+/Emma’s Diary/Baby 2 Body/Oviva/Baby Buddy/Other	12 (41%)
**Leaflet**	28 (23%)	23 (19%)	NHS leaflets	18 (78%)
			Local hospital trust	13 (57%)
			British Dietetic Association/Royal College of Midwives/Royal College of Obstetricians and Gynaecologists	7 (4%)

Percentages do not add up to 100% due to participants choosing more than one category

^a^Some categories have been merged due to low values

^b^Handheld records may include nutrition/diet information as leaflets or pamphlets

## Discussion

In general, midwives were not confident in their ability to provide guidance to women on foods and drinks to avoid or limit, with > 90% reporting that were *Confident/Very confident* for only four items: liver/liver products, unpasteurised milk, alcohol and eggs. There were some items for which they were particularly unconfident (< 65% *Confident/Very confident* for herbal tea, omega-3 supplements, game birds and game meat). Although most midwives reported being *Confident/Very confident* in their knowledge of the guidance on fish (> 70% for all items related to fish), their recall for most of these items was usually inaccurate (for example, 38% correct recall for guidance on tinned tuna, 53% for oily fish). Similarly for gamebirds and game meat, recall was correct in only 31% and 26%, respectively. Since the most usual method of delivery of information was verbal it is essential that midwives are able to recall the information confidently and accurately, and it suggests that there is a need for further training for midwives to support their knowledge. Nearly 20% of midwives reported that they were never able to deliver the ‘level of service that they would like to’ on information on diet in pregnancy, and this was primarily due to lack of time in appointments and lack of suitable and accessible training.

Lack of confidence in knowledge on the restriction advised on herbal tea (no more than four cups per day [3]) is particularly concerning. Herbal teas commonly used by women during pregnancy include ginger, raspberry, cranberry, echinacea, peppermint and chamomile [38], and they have traditionally been used to treat a range of conditions including nausea, anaemia, constipation, heartburn and sleeping problems, and used in preparation for labour. Herbal teas carry a risk of herb–drug interactions: for example, tannin-containing herbs, such as raspberry leaf, can interfere with iron absorption; ginger and chamomile enhance the effect of CNS depressants so are not advised in patients taking drugs such as clonazepam; St John’s wort interacts with several drugs (for example anticoagulants, anticonvulsants, immunosuppressants). Other herbs contra-indicated in pregnancy include cohosh, Ginko bilbao, St John’s wort and others, because they are abortifacients, cathartic laxatives or emmenagogues, or have hormonal effects or affect uterine contractions [38]. There is recent evidence that women simultaneously cut back on caffeinated drinks as advised but increase herbal tea consumption [33], possibly as a result of herbal teas being seen as a ‘healthy choice’ [39].

Lead-shot game meat and gamebirds are likely to be consumed by few women, but amongst that those that do consume them, it is likely that some women will be frequent consumers [40] (for example, women who have a personal or professional association with shooting activities). Midwives need to be aware of both this group of women and the guidance on lead-shot game meat/gamebirds in order to deliver information to this group of women, with aim of preventing adverse neurodevelopmental outcomes in the fetus [10]. The identification of meat as not being lead shot, perhaps through a voluntary labelling scheme, would be helpful in this regard.

For the items for which midwives were asked to recall the guidance, poor knowledge around frequently consumed types of fish, such as tinned tuna, was striking. Many respondents recalled the advice as being up to two medium-sized cans of tuna per week, when it is actually up to four cans per week [3]. This may be caused by confusion with the population level message to eat at least two portions of fish per week (including during pregnancy) [2, 31]. There was similar uncertainty about the number of portions of oily fish advised (the correct advice is to eat at least one but no more than two portions a week [3]).

Healthcare professionals, including midwives, have been identified in other studies as being trusted providers of this information by women [25, 29], and they have a central role in health promotion facilitated by regular contact with women throughout their pregnancy [41]. In Australia, it has been observed that most interactions between midwives and women are medically directed [42], and this is likely to be similar in England, limiting opportunities for discussion of diet-related information. In a narrative review of the role of midwives in the promotion of healthy lifestyle in pregnancy, Bahri Khomami et al. [22] identified several barriers to optimal delivery of information by midwives, including lack of content in undergraduate midwifery curricula to provide the knowledge, skills and confidence needed to assess and support healthy lifestyles, and lack of training in the workplace. A systemic review specifically of nutrition advice in pregnancy reported similar barriers in healthcare professionals in many countries including the UK, Europe, Australia, New Zealand, Canada and the United States of America [43]. For midwives in the present study the barriers to delivering guidance on foods/drinks to avoid or limit also included lack of training and lack of access to suitable resources; in addition the participants highlighted the lack of time in appointments, which were largely focused on clinical aspects. For women, barriers to following advice on foods/drinks to avoid or limit may include lack of awareness, but also having had no prior illness from consuming those foods, preferences for those foods, perception of their health benefits, and convenience [44]. As stated by Bahri Khomami et al. [22], it is critical that barriers to the provision of best practice by midwives at all levels (individual, system and policy) are addressed, including provision of undergraduate and postgraduate training, and enabling health systems to include adequate appointment time to enable provision of information for women within standard care. Co-designed training materials and resources for midwives could encompass, for example, annual professional training courses, intermittent nutrition specialism courses, and online resources.

Where women lack knowledge on guidance on foods and drinks to avoid and limit, they will continue to consume foods and drinks that could put them and their fetus at risk of adverse pregnancy outcomes (in Australia, for example, 83% of a sample of 223 women incorrectly identified at least one unsafe food as being safe to eat [27]; similarly, Canadian women’s knowledge of foods that are high-risk for listeria was poor [25]). This strengthens the case for the role of healthcare providers, including midwives, in providing or signposting information. However, even knowledge of the guidance does not always result in women following it: a study of recently postpartum women in Ireland found that even though more than 80% of the sample (*n* = 271) knew that they should avoid foods at high risk of transmitting listeria during their pregnancy, 55% reported consuming them during their pregnancy [26]. Although women are known to have a high rate of compliance with guidance on health-related behaviours such as cigarette smoking and alcohol consumption in pregnancy, changes related to heathy eating such as increases in fruit and vegetable intake are harder to achieve [45], with little change in dietary patterns from before to during pregnancy [46]. However, for foods and drinks to avoid or limit where is a clear association between consumption and a possible hazard, risk aversion emerges as an additional factor that may be powerful in altering consumption patterns: for example, risk aversion to mercury underpinned other themes that together shaped perception of fish consumption during pregnancy in a qualitative study in Australia [24]. This suggests that both the content and delivery of information may need to be designed in a different way from healthy eating information in order to have an impact.

The NHS website was also identified as a key source of information that midwives signposted women to. Midwives should be enabled to be familiar with the information there and have an accurate recall of it, and the website should provide clear, up-to-date evidence-based guidance. The guidance on fish, for example, does not include the overarching message for pregnant women to eat at least two portions of fish a week as recommended by SACN [31], and this may contribute to fish intakes in pregnancy being below those recommended [3, 47]. Updates (for example, new guidance on cooking smoked fish in response to a listeria outbreak linked to smoked fish in 2022) should be well publicised to both women and midwives. Advice on omega-3 supplements is currently displayed in on a page concerned with supplements rather than the main page [5]: this could be made more readily accessible.

The strengths of this study are twofold. First, we used the Think Aloud process to validate the questionnaire with participants who fulfilled the eligibility criteria for the finalised questionnaire. Second, reporting bias was minimised as the anonymity of the online survey method enabled midwives to be honest in stating whether they felt unconfident in their knowledge in a way that they might not have been in a group or even a one-to-one setting.

There are several limitations to this study. First, we only included 122 respondents, although this was sufficient to indicate areas of uncertainty in their knowledge and the need for further in time appointments and specialist training. However, it prevented high powered analysis of the associations between demographic characteristics of the midwives and their responses. Our survey was open during Covid restrictions, which had a great impact on the daily workload of midwives, and this may have limited the response rate. Second, the study was not representative of the population of midwives in England, particularly in geographical location and ethnicity, which limits generalisability. Third, we were unable to include questions about recall for all foods/drinks for which guidance is to avoid or limit, and this will important to include in future larger scale studies. Finally, many midwife-led appointments with women were conducted online during this time and this may have affected time available and the clarity of communication between midwives and pregnant women compared with usual face-to-face appointments.

## Conclusion

In conclusion, midwives were not confident about many foods and drinks to avoid or limit in pregnancy, and there were some items for which they were mistaken when asked to recall the guidance. Midwives would like to be able to access appropriate training and resources on the guidance on foods and drinks to avoid or limit, and have adequate time available in appointments to discuss this. As the main source of information that midwives signpost to women, is important that the NHS website is completely clear and consistent in its messages. Further research is needed on the effectiveness of the guidance through exploration of the barriers and enablers to the optimal implementation of the guidance by pregnant women.
